# RNS60, a charge-stabilized nanostructure saline alters *Xenopus Laevis* oocyte biophysical membrane properties by enhancing mitochondrial ATP production

**DOI:** 10.14814/phy2.12261

**Published:** 2015-03-05

**Authors:** Soonwook Choi, Eunah Yu, Duk-Soo Kim, Mutsuyuki Sugimori, Rodolfo R Llinás

**Affiliations:** 1 Marine Biological LaboratoryWoods Hole, Massachusetts, USA; 2 Department of Neuroscience and Physiology, New York University School of MedicineNew York, New York, USA; 3 Department of Anatomy, College of Medicine, Soonchunhyang UniversityCheonan-Si, Korea

**Keywords:** Adenosine triphosphate, membrane potential, *Xenopus laevis*

## Abstract

We have examined the effects of RNS60, a 0.9% saline containing charge-stabilized oxygen nanobubble-based structures. RNS60 is generated by subjecting normal saline to Taylor–Couette–Poiseuille (TCP) flow under elevated oxygen pressure. This study, implemented in *Xenopus laevis* oocytes, addresses both the electrophysiological membrane properties and parallel biological processes in the cytoplasm. Intracellular recordings from defolliculated *X. laevis* oocytes were implemented in: (1) air oxygenated standard Ringer's solution, (2) RNS60-based Ringer's solution, (3) RNS10.3 (TCP-modified saline without excess oxygen)-based Ringer's, and (4) ONS60 (saline containing high pressure oxygen without TCP modification)-based Ringer's. RNS60-based Ringer's solution induced membrane hyperpolarization from the resting membrane potential. This effect was prevented by: (1) ouabain (a blocker of the sodium/potassium ATPase), (2) rotenone (a mitochondrial electron transfer chain inhibitor preventing usable ATP synthesis), and (3) oligomycin A (an inhibitor of ATP synthase) indicating that RNS60 effects intracellular ATP levels. Increased intracellular ATP levels following RNS60 treatment were directly demonstrated using luciferin/luciferase photon emission. These results indicate that RNS60 alters intrinsic the electrophysiological properties of the *X. laevis* oocyte membrane by increasing mitochondrial-based ATP synthesis. Ultrastructural analysis of the oocyte cytoplasm demonstrated increased mitochondrial length in the presence of RNS60-based Ringer's solution. It is concluded that the biological properties of RNS60 relate to its ability to optimize ATP synthesis.

## Introduction

RNS60, a physically modified saline containing charge-stabilized oxygen nanobubbles, is generated by subjecting normal saline (0.9% sodium chloride, USP pH5.6) to a noncentrifuge rotor/stator device, which incorporates controlled turbulence and Taylor–Couette–Poiseuille (TCP) flow under elevated oxygen pressure (1 atm of oxygen back-pressure) at 4°C (Hamakawa et al. 2008; Khasnavis et al. 2012). The gas/liquid interface and energy transfer are maximized using a rotor with numerous cavities and rotational speeds above 3450 rpm. These conditions generate a strong shear layer at the interface between the vapor and liquid phases near the rotor cavities, which correlates with the generation of small bubbles from cavitation (Hamakawa et al. 2008). Chemically, RNS60 contains water, sodium chloride, 55 ± 5 ppm oxygen, but no active pharmaceutical ingredients.

Although there are no active pharmaceutical ingredients, it has been proposed that RNS60 may be developed as a therapeutic intervention for neuroinflammatory and neurodegenerative disorders. Both in cellular assays and MPTP-intoxicated mice, an animal model of Parkinson's disease, treatment of RNS60 has demonstrated robust anti-inflammatory and neuroprotective properties (Khasnavis et al. 2012, 2014). Also, RNS60 treatment resulted in an ameliorated adoptive transfer of experimental allergic encephalomyelitis in an animal model of multiple sclerosis (Mondal et al. 2012).

Although RNS60 has demonstrated a significant improvement in neurodegenerative and respiratory diseases, and neuronal plasticity, RNS60's precise mechanism of action is still under active investigation. Recently, we showed that RNS60 positively modulates synaptic transmission by upregulating ATP synthesis, leading to synaptic transmission enhancement (Choi et al. 2014). In this study we further address the effect of RNS60 on the intrinsic membrane properties and investigate the underlying cellular mechanisms using *Xenopus laevis* oocytes.

## Materials and Methods

### Intracellular recording

Intracellular recordings were obtained from defolliculated *Xenopus laevis* oocytes (Ecocyte Bioscience US LLC, Austin, TX) using glass micropipettes filled with 3M potassium acetate (1–2 MΩ). The database for these experiments included oocytes that had a stable resting potential of at least −30 mV for at least 5 min after the introduction of electrodes. Oocytes were superfused with a freshly made solution containing 115 mmol/L NaCl, 2 mmol/L KCl, 1.8 mmol/L CaCl_2_, and 5 mmol/L HEPES. Voltage levels were recorded using a dual channel amplifier (Neuro Data Instruments Corp., New York, NY), and analyzed using a Digidata 1440 (Molecular device) with pCLAMP software (version 10.2, Molecular device). Cell input resistance was determined as the ratio of the steady-state voltage change during small transmembrane current pulses. To block the activity of Na^+^/K^+^ ATPase, a stock solution of 100 mmol/L ouabain (×1000) was prepared in DMSO, the stock solution was diluted with recording solution to obtain a final concentration of 100 *μ*mol/L ouabain in the recording chamber.

### ATP synthesis measurements using luciferin/luciferase

ATP synthesis was directly measured using luciferin/luciferase light emitting measurements (McElroy 1947; Strehler and Mc 1949). Luciferase (<50 nL of 5 *μ*mol/L) was directly injected into *X. laevis* oocytes and luciferin was added to bath solution (final concentration of luciferin in recording solution, 200 *μ*g/mL). Light emission was monitored and imaged using a single-photon counting video camera (Argus −100, Hamamatsu Photonics). Light magnitude was determined in photon images of ATP obtained by the accumulation of illuminated photon for 1 min.

### Ultrastructural analysis

*Xenopus laevis* oocytes were prepared according to the standard procedures for TEM (transelectron microscopy) as described previously (Allen et al. 2007). Briefly, immediately following a 5-min incubation period in RNS60-based Ringer's solution or standard Ringer's solution, oocytes were fixed by immersion in 2% glutaraldehyde. The oocytes were then postfixed in osmium tetroxide (OSO_4_), stained in block with uranium acetate, dehydrated, and embedded in resin (Embed 812, EM Sciences). Seventy-nanometer-thick ultrathin sections were collected on Formvar/Carbon on 200 mesh grids, and contrasted with uranyl acetate and lead citrate. Images were captured using an H-7500 TEM (Hitachi High-Tech, Tokyo, Japan) at 2100×, 7000×, and 21,000×, magnification, respectively.

## Results

### Electrophysiological membrane properties of *Xenopus laevis* oocytes are modified by RNS60-based Ringer's

Intracellular recordings from defolliculated *X. laevis* oocytes were obtained in four different solutions: RNS60-based Ringer's, RNS10.3 (TCP-modified saline without added oxygen)-based Ringer's, ONS60 (saline containing comparable level of oxygen as RNS60 without the TCP modification)-based Ringer's, and air-exposed normal Ringer's solution.

**Figure 1:**
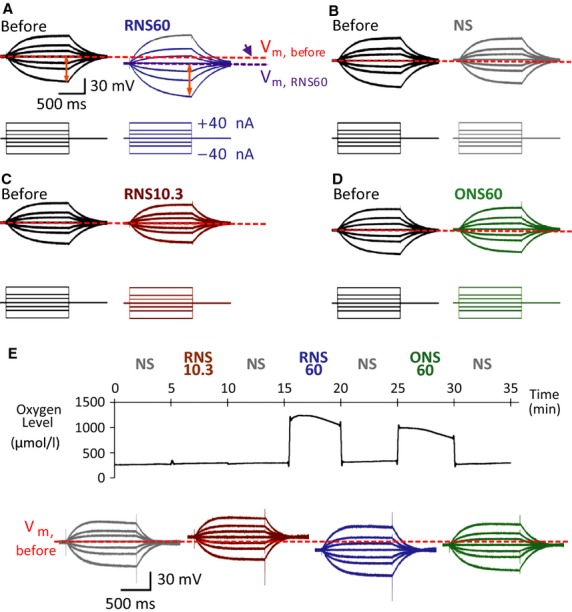
Intracellular recordings from *Xenopus laevis* oocytes in the presence of RNS60- (A), NS- (B), RNS10.3- (C), and ONS60- (D)-based Ringer's solutions. Note that solutions based on RNS60, but not those based on normal saline (NS), RNS10.3 (TCP-modified saline without excess oxygen), or ONS60 (saline containing comparable level of oxygen without TCP modification), increased (hyperpolarized) resting membrane potential. Also, step pulse transmembrane current injection-induced membrane potential changes were increased only in the presence of RNS60. (E) Intracellular recording with serial applications of NS, RNS10.3, RNS60, or ONS60 with measurement of oxygen level in recording solutions.

As shown in Figure1A, RNS60 (but not NS, RNS10.3, or ONS60, B–D, respectively) increased the resting membrane potential by −6.5 mV ± 1.0 in 16 *X. laevis* oocytes (blue arrow in Figure1A and statistical measurements in Table1). In the serial applications of normal Ringer's or RNS10.3-, RNS60-, and ONS60-based Ringer's, the membrane potential was increased only after administration of RNS60-based Ringer's solution (Fig.1E). In parallel with the increased membrane potential, RNS60 significantly increased the membrane input resistance from 1.11 ± 0.04 MΩ to 1.41 ± 0.08 MΩ (Table1 and Fig.1A, red arrows).

**Table 1:** The effects of RNS60 on membrane potential (*V*_m_) and membrane input resistance (*R*_m_) of *Xenopus laevis* oocytes

	RNS60 (16)	NS (13)	RNS10.3 (14)	ONS60 (10)
*V*_m_ (mV)	−47.2 ± 2.0	−42.8 ± 2.2	−43.1 ± 1.8	−41.1 ± 1.5
Δ*V*_m_ (mV)	−6.5 ± 1.0***	−1.9 ± 1.3	−1.7 ± 1.0	−0.6 ± 1.0
*R*_m_ (MΩ)	1.41 ± 0.08	1.18 ± 0.09	0.93 ± 0.04	1.02 ± 0.08
Δ*R*_m_ (MΩ)	0.31 ± 0.07**	0.02 ± 0.04	−0.08 ± 0.03	−0.04 ± 0.02

Data are mean ± SEM and number in parenthesis is number of *X. laevis* oocytes analyzed.

Note that: RNS60 hyperpolarized the resting membrane potential and increased the membrane input resistance (**P < 0.01 and ***P < 0.001 by paired *t*-test).

**Figure 2:**
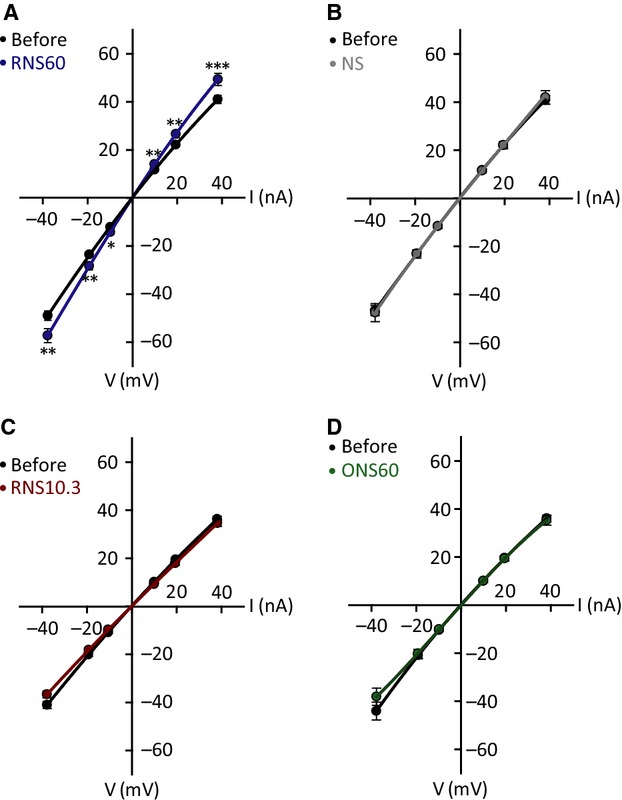
Membrane potential changes as a function of current injection (I–V curve) in the presence of RNS60 (A, average of 16 experiments), NS (B, average of 13 experiments), RNS10.3 (C, 14 experiments) and ONS60 (D, 10 experiments). *P <* *0.05, **P <* *0.01 and ***P <* *0.001 two-way ANOVA/Tukey's post-hoc test.

Plotting the current voltage relationship, under the different conditions described above, generates a more complete picture of the electrophysiological changes in membrane properties observed with the different superfusion fluids utilized. This is illustrated in Figure2. Because the intrinsic electrophysiological membrane properties of the oocytes recorded using ONS60, RNS10.3, and NS were indistinguishable (Figs.1, 2), NS was used as the control solution in the experiments in Figures 4–6.

### RNS60 increases membrane potential by increasing Na/K-ATPase activity

Plasmalemal Na^+^/K^+^-ATPase significantly contributes to the oocyte membrane potential via an ouabain-sensitive current (Lafaire and Schwarz 1986). In order to determine whether RNS60-induced membrane hyperpolarization is Na^+^/K^+^-ATPase dependent, we examined the effect of RNS60 on membrane potential following Na^+^/K^+^-ATPase block with 100 *μ*mol/L ouabain.

**Figure 3:**
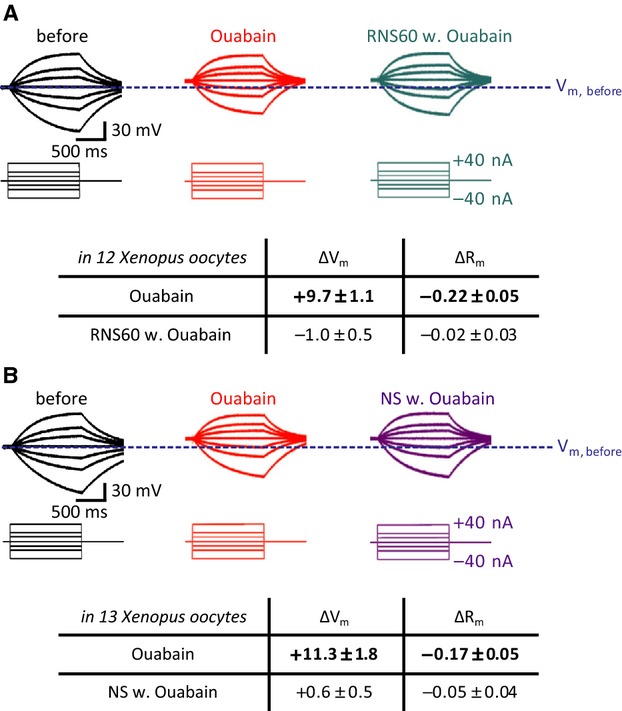
RNS60 effect on *Xenopus laevis* oocyte membrane potential and input resistance following ouabain Na^+^/K^+^ ATPs block. In (A) Control, left set of recordings (in black), resting potential and input resistance recordings before ouabain administration. In red similar set of recordings 10 min following 100 *μ*mol/L ouabain administration. The oocyte resting potential was reduced by nearly 10 mV (*P *<* *0.001 by paired *t*-test). In addition, a significant decrease in membrane input resistance was also registered (*P *<* *0.05 by paired *t*-test). In green, following ouabain addition of RNS60 did not modify *X. laevis* oocyte resting potential or membrane resistance (results from 13 different oocyte experiments). In (B) a similar set of recordings as in (A). The last set in purple demonstrates no change of membrane properties in normal saline solution with ouabain (results from 10 different oocyte experiments).

Thus, as shown in Figure3, RNS60-dependent membrane hyperpolarization effects are completely gone in the presence of ouabain. Furthermore, following initial ouabain-induced membrane depolarization, RNS60 failed to recover initial membrane potential.

### RNS60 membrane hyperpolarization is triggered by increased intracellular ATP via mitochondrial activation

The next issue addressed related to whether the effects of RNS60 on membrane potential, triggered through activation of Na^+^/K^+^-ATPase, were directly related to an intracellular ATP increase. This mechanism was reported for membrane potential increase in squid giant synapse (Choi et al. 2014). In that case, the source of energy that Na^+^/K^+^-ATPase uses for the Na^+^, K^+^ exchange (Xie and Askari 2002) was ATP.

**Figure 4:**
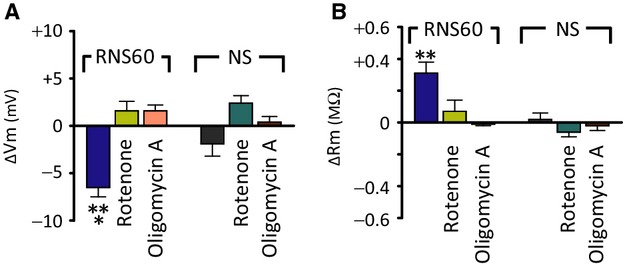
Effects of RNS60 on membrane potential (A) and membrane input resistance (B) following block of intracellular “usable ATP” inhibition. Note that RNS60 effect is absent after both the inhibition of electron transport chain in mitochondria by rotenone and after oligomycin A, an inhibitor of ATP synthase. **P <* *0.01 and ***P <* *0.001 by paired *t*-test, indicating that the target for RNS60 action is mitochondrial ATP synthesis (*n* = 7 in RNS60 with rotenone, 6 in RNS60 with oligomycin A, 8 in NS with rotenone, and 6 in NS with oligomycin A).

As shown earlier for the squid synapse (Choi et al. 2014), RNS60-induced changes of intrinsic properties of oocyte membrane were prevented both by 500 nmol/L Rotenone and 2.5 mg/mL oligomycin A (Fig.4). Rotenone is an inhibitor of the electron transport chain in mitochondria and oligomycin A, an inhibitor of ATP synthase that blocks the production of “usable” intracellular ATP. Indeed, as expected, RNS60-induced hyperpolarization was prevented following the application of rotenone or oligomycin A, indicating that the membrane hyperpolarizing effect of RNS60 was dependent on intracellular ATP level.

### Imaging intracellular ATP level

**Figure 5:**
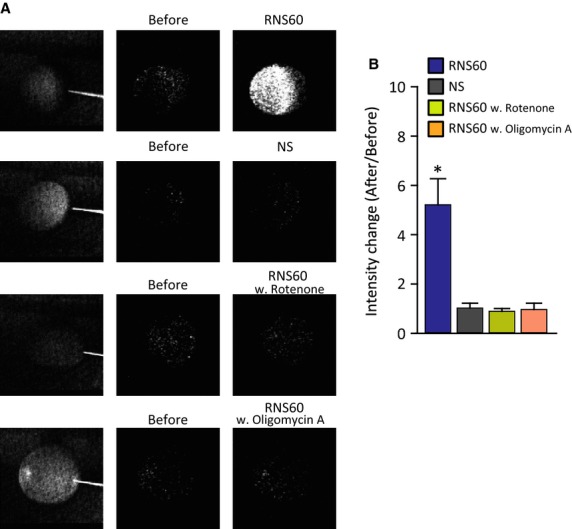
Intracellular ATP levels determined by luciferin/luciferase photon emission in *Xenopus laevis* oocytes. ATP levels increased 5 min following RNS60-based Ringer's superfusion, but not so following RNS-based Riger's with intracellular ATP blockages (with rotenone and oligomycin A) as well as NS-based Ringer's superfusion (A). Photon ATP images were obtained by photon accumulation for 1 min. Scale bar = 0.5 mm. Statistical difference is shown in (B). *P <* *0.05 by one-way ANOVA. (*n* = 8 in RNS60, 6 in NS, 4 in RNS60 with rotenone, and 5 in RNS60 with oligomycin A).

Intracellular ATP levels were then directly determined using ATP-dependent luciferin/luciferase (L/L) photon emission to examine the level to which RNS60 actually increases intracellular ATP. As shown in Figure5, L/L fluorescence intensity was significantly increased following RNS60-based Ringer's introduction as the bathing solution for 5 min, compared that in control Ringer's solution. The increase of intracellular ATP after RNS60 application to *X*. oocytes was prevented by both 500 nmol/L Rotenone and 2.5 mg/mL oligomycin A (Fig.5), suggesting that the effect of RNS60 was related to the alteration of intracellular ATP levels.

### Ultrastructural changes in mitochondrial morphology following RNS60 superfusion

**Figure 6:**
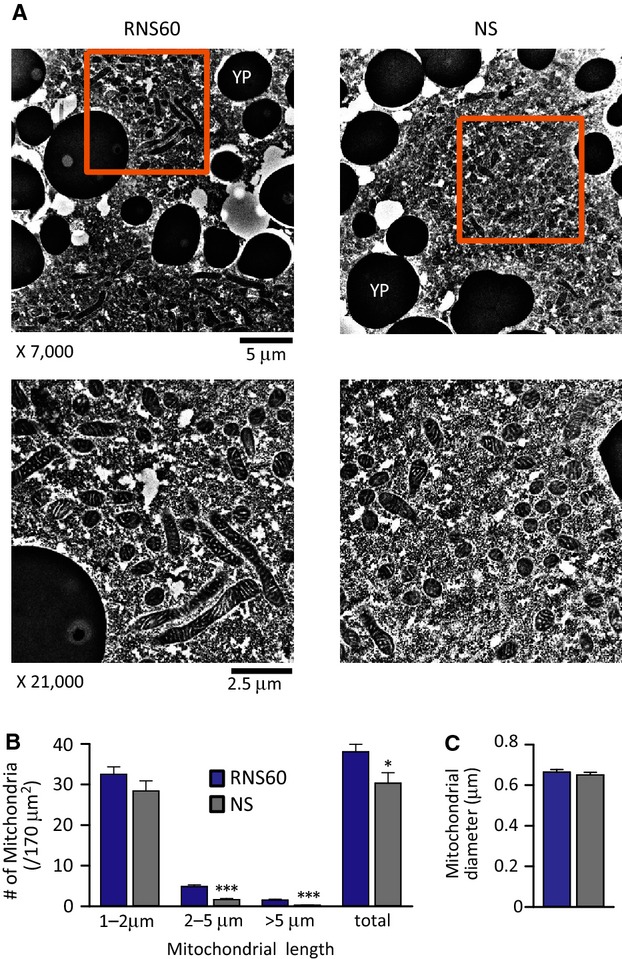
Ultrastructural analysis of mitochondria in response to RNS60-based Ringer's, compared to air oxygenated standard NS-based Ringer's. (A) Electron microscopy of cortical cytoplasm in *Xenopus laevis* oocytes. (B) The increased number of longer length mitochondria in response to RNS60. Total 36 areas (area size: 170 μm^2^, 6 areas per *X*. *laevis* oocyte) in cortical cytoplasm were analyzed in each group, YP: light yolk platelet. (C) The comparison of averaged mitochondrial diameter. Statistically significant differences marked as *P <* *0.05 and ***P <* *0.001 by one-way ANOVA.

Ultrastructural analysis of cortical cytoplasm in oocytes bathed for 5 min in control or RNS60-based Ringer's demonstrated clear and statistically significant changes in mitochondrial ultrastruture. In RNS60-based Ringer's solution, electron-microscopical analysis demonstrated an average increase of mitochondrial length compared with the mitochondrial length in normal saline solution (Fig.6A). Indeed, the number of mitochondria having >2 *μ*m length was significantly increased (Fig.6B).

## Discussion

The present set of electrophysiological, photoimaging, and ultrastructural results clearly indicate that RNS60 has a distinct effect on both the resting and voltage-dependent electrical membrane properties, as well as on the level of ATP generation and the size of mitochondrial elements, in the *Xenopus laevis* oocyte.

### Concerning the mechanism by which the input resistance and I-V curve were modified following RNS60 superfusion

The voltage-sensitive properties of *X. laevis* oocyte membranes activated by square pulse current injection have been amply studied by previous investigators in the field (Baud et al. 1982; Dascal et al. 1984). This, plus the fact that these oocytes present robust and stable biophysical membrane characteristics, makes this preparation ideal for determining possible modification of biophysical membrane properties. Membrane potential changes following negative current pulses are larger than those produced by positive current injection, in accordance with the outward rectification properties of *X. laevis* oocytes in response to positive current injection (Baud et al. 1982; Bauer et al. 1996). Because RNS60 increases membrane potential and input resistance, the changes in response to current injection may themselves be modified as a result of resting membrane potential modification. This itself may reduce the rectification properties of the membrane.

It has been shown, however, that the relationship between resting membrane potential and input resistance is linear in the oocyte membrane (Dascal et al. 1984) and that input resistance of oocyte membrane is increased at hyperpolarized membrane potentials. This being the case, the change of input resistance and I-V curve after RNS60 application may be the result of an increased membrane potential induced by RNS60.

### Concerning the relationship between the intracellular ATP level and membrane potential

It has been shown that the intracellular ATP is released through activated hemichannels in *X. laevis* oocytes (Bahima et al. 2006). In renal epitheloid MDCK-cells, ATP leads to membrane hyperpolarization (Paulmichl et al. 1991). In addition, application of ATP produced the initial hyperpolarization in smooth muscle cells of mouse uterus (Ninomiya and Suzuki 1983). This being the case, the RNS60-induced increase of intracellular ATP may be the direct reason behind the induced membrane hyperpolarization in *X. laevis* oocytes.

### Concerning RNS60 effect on mitochondrial ultrastructure

The electronmicroscopical findings presented here were assembled from the “Mitochondrial cloud” (Balbini Body) (Heasman et al. 1984). The results indicate an increase in both mitochondrial size and ATP production that is in line with the electrophysiological findings concerning oocyte optimization membrane properties.

Although the relationship between the size and the dynamics of mitochondria is complex, mitochondrial volume has been shown to correlate positively with the efficiency of mitochondrial function (Bach et al. 2003). In the mitochondria of brown adipose tissue, moreover, morphometric analysis revealed that the increased mitochondrial size could be attributed to enhance mitochondrial activity (Lagouge et al. 2006). Conversely, it has also been shown that the reduction of mitochondrial size was associated with a reduction in steady-state ATP levels (Nisoli et al. 2004). Thus, it is likely that the increase of mitochondrial size is a morphological correlate of the increased mitochondrial function in the *X. laevis* oocytes treated with RNS60.

Because of the importance of mitochondrial energy production in embryogenesis, numerous studies have investigated number and size of mitochondria in *X. laevis* oocytes over the last four decades (Marinos and Billett 1981; Heasman et al. 1984; Dumollard et al. 2007). Based on our data, it is tempting to propose that embryogenesis may be optimized by the oxygen-containing nanostructures in RNS60.
